# Supplementary material for: Mechanisms governing the pioneering and redistribution capabilities of the non-classical pioneer PU.1
Source: Nat Commun. 2020 Jan 21;11:402. doi: 10.1038/s41467-019-13960-2 (PMC6972792; doi:10.1038/s41467-019-13960-2)
Supplement: Supplementary file 7 — Source data [file 41467_2019_13960_MOESM7_ESM.zip › Source_Data/Figure6/Figure6B_MotifScanOutput/FLI1_PU.1/homerResults/motif15.similar.html]

motif15

## Information for motif15

A
G
T
C
C
A
T
G
T
C
G
A
A
C
G
T
A
C
T
G
C
G
T
A
T
A
G
C
A
C
G
T
A
T
G
C
C
G
T
A
A
G
T
C
  
Reverse Opposite:  

T
C
A
G
A
C
G
T
A
T
C
G
C
G
T
A
A
T
C
G
A
C
G
T
T
G
A
C
C
G
T
A
A
G
C
T
G
T
A
C
T
C
A
G
  

|  |  |
| --- | --- |
| p-value: | 1e-105 |
| log p-value: | -2.420e+02 |
| Information Content per bp: | 1.663 |
| Number of Target Sequences with motif | 728.0 |
| Percentage of Target Sequences with motif | 7.32% |
| Number of Background Sequences with motif | 874.2 |
| Percentage of Background Sequences with motif | 2.40% |
| Average Position of motif in Targets | 95.9 +/- 53.2bp |
| Average Position of motif in Background | 101.5 +/- 64.2bp |
| Strand Bias (log2 ratio + to - strand density) | -0.0 |
| Multiplicity (# of sites on avg that occur together) | 1.04 |
| Motif File: | file (matrix) reverse opposite |

### Similar de novo motifs found

|  |  |  |  |  |  |  |  |
| --- | --- | --- | --- | --- | --- | --- | --- |
| Rank | Match Score | Redundant Motif | P-value | log P-value | % of Targets | % of Background | Motif file |
| 1 | 0.902 | T G C A A C G T A C T G G T C A C T A G A C G T G T A C C T G A A G C T | 1e-103 | -237.475490 | 7.23% | 2.38% | motif file (matrix) |
| 2 | 0.977 | T C G A A C G T A T C G G C T A A T C G A C G T G T A C C T G A A G C T G A C T | 1e-102 | -237.049770 | 7.87% | 2.76% | motif file (matrix) |
| 3 | 0.957 | A C G T A C T G G T C A T A C G A C G T G T A C C T G A A G C T | 1e-97 | -225.085940 | 10.00% | 4.21% | motif file (matrix) |
| 4 | 0.952 | C A G T C T A G T G A C A C G T A C T G C G T A A T C G A C G T A G T C C G T A A G C T G A C T | 1e-93 | -215.621485 | 6.20% | 1.95% | motif file (matrix) |
| 5 | 0.922 | A C G T A C T G C G T A A C T G A C G T A G T C C G T A | 1e-90 | -207.995517 | 7.88% | 3.01% | motif file (matrix) |
